# Biliverdin regulates NR2E3 and zebrafish retinal photoreceptor development

**DOI:** 10.1038/s41598-022-11502-3

**Published:** 2022-05-04

**Authors:** Blaine Connor, Kayla Titialii-Torres, Abigail E. Rockenhaus, Samuel Passamonte, Ann C. Morris, Young-Sam Lee

**Affiliations:** 1 Department of Biology, Johns Hopkins University, Baltimore, MD 21218 USA; 2 Department of Biology, University of Kentucky, Lexington, KY 40506 USA; 3 Department of Molecular and Cellular Biochemistry, University of Kentucky College of Medicine, Lexington, KY 40536 USA

**Keywords:** Transcription factors, Zebrafish

## Abstract

NR2E3 is an orphan nuclear receptor whose loss-of-function causes abnormal retinal photoreceptor development and degeneration. However, despite that many nuclear receptors are regulated by binding of small molecule ligands, biological small molecule ligands regulating NR2E3 have not been identified. Identification of an endogenous NR2E3 ligand might reveal a previously unrecognized component contributing to retinal development and maintenance. Here we report that biliverdin, a conserved green pigment from heme catabolism, regulates NR2E3 and is necessary for zebrafish retinal photoreceptor development. Biliverdin from retinal extracts specifically bound to NR2E3’s ligand-binding domain and induced NR2E3-dependent reporter gene expression. Inhibition of biliverdin synthesis decreased photoreceptor cell populations in zebrafish larvae, and this phenotype was alleviated by exogenously supplied biliverdin. Thus, biliverdin is an endogenous small molecule ligand for NR2E3 and a component necessary for the proper development of photoreceptor cells. This result suggests a possible role of heme metabolism in the regulation of retinal photoreceptor cell development.

## Introduction

NR2E3, also known as photoreceptor-specific nuclear receptor (NR) or PNR, is a transcription factor enriched in developing and adult retina ^1^. Mutations in the *NR2E3* gene were found to cause inherited human diseases like Enhanced S-Cone Syndrome (ESCS) and retinitis pigmentosa 37 (RP37)^1–3^. These diseases are characterized by fewer rod photoreceptor cells with an increased number of short wavelength sensitive cone-like cells, suggesting a role of NR2E3 in retinal photoreceptor cell development and maintenance. Indeed, NR2E3, together with other retinal transcription factors like Crx, Nrl, and NR1D1, contributes to the regulation of photoreceptor-specific gene expression^4–6^. These functions of NR2E3 were also observed in other vertebrates like frogs and zebrafish^7,8^.

NR2E3 has also been suggested as a potential target to treat retinal disorders^9,10^. Forced expression of NR2E3 was shown to suppress the progression of retinitis pigmentosa in several mouse models^11^, while a synthetic NR2E3 antagonist suppressed the progression of retinitis pigmentosa caused by mutated rhodopsin^12^.

NR2E3 has a domain architecture shared by most other NRs^13^ (Figure S1A). It has an amino-terminal unstructured region harboring a trans-activator motif (AF1), followed by zinc-finger DNA-binding domain (DBD), a hinge region, and a carboxy-terminal ligand-binding domain (LBD) that harbors the second transactivation helix AF2. In the case of better characterized NRs, binding of small molecule ligand to LBD is a major regulatory mechanism^13^. Considering that a synthetic NR2E3 antagonist regulates NR2E3-dependent gene expression in cells and in animals^12^, we suspected that there might be a retinal small molecule regulating NR2E3 in vivo. At this moment, an endogenous NR2E3 ligand has not been identified, and NR2E3 remains as an orphan NR. Identification of an endogenous NR2E3 ligand might reveal a novel component contributing to retinal development and maintenance.

Here we report that biliverdin is an endogenous ligand regulating NR2E3 and is a compound that contributes to the development of zebrafish retina. Biliverdin is a conserved green pigment synthesized from heme by heme oxygenase (HO) isozymes^14^. HO isozymes are expressed in retina and other tissues, and their expressions are induced by light exposure^15^. Biliverdin is reduced to bilirubin by biliverdin reductase (BVR) isozymes^16^. We found that biliverdin from retinal extract specifically bound to NR2E3’s ligand-binding domain (NR2E3^LBD^) in vitro. We also found that biliverdin induced NR2E3-dependent reporter gene expression in cells. Pharmacological inhibition of biliverdin synthesis in zebrafish larvae decreased the population of photoreceptor cells, and this phenotype was alleviated by exogenously supplied biliverdin. These results indicated that biliverdin is an endogenous small molecule regulating NR2E3 and a component contributing to the proper development of retinal photoreceptor cells. Together with NR1D1 that is regulated by heme^17,18^, this role of biliverdin suggests a connection between heme metabolism and retinal development.

## Results

### Identification of biliverdin’s interaction with NR2E3^LBD^ in vitro

**Figure 1:**
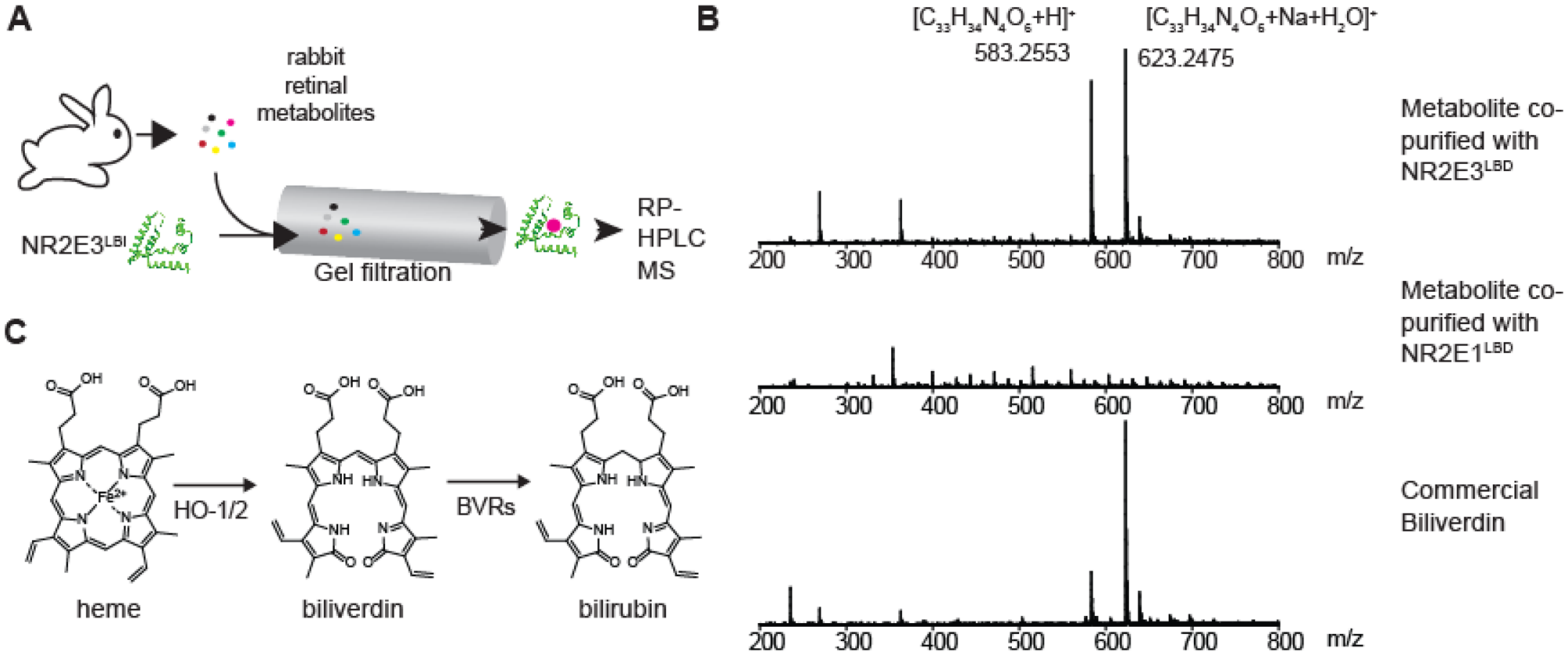
Identification of biliverdin as a potential NR2E3^LBD^ ligand. (**A**) A schematic representation of the method. Small molecules extracted from rabbit retina were incubated with recombinant MBP-NR2E3^LBD^ expressed and purified from *E. coli*. Protein-bound small molecules were isolated by a gel filtration chromatography and identified by mass spectrometry. (**B**) Mass spectra (ESI, positive ions) of metabolites co-purified with MBP-NR2E3^LBD^, ones eluted in the gel filtration fraction without any protein, and commercial biliverdin. (**C**) A schematic representation of biliverdin and its metabolism (HO-1/2: heme oxygenases 1 and 2, BVRs: biliverdin reductases).

To gain insight into what might regulate NR2E3 in retina, we searched for a retinal small molecule specifically binding to NR2E3^LBD^. For this purpose, we used a combination of gel filtration chromatography and mass spectrometry^19,20^. Recombinant maltose-binding domain (MBP)-NR2E3^LBD^ (amino acid residues 164–410 of 410 residue NR2E3) fusion protein was expressed and purified from *E. coli* as in literature^21^. As previously reported^21^, without the MBP-tag, purified NR2E3^LBD^ was prone to aggregation. We thus used MBP-NR2E3^LBD^ without removing the tag. Retinal small molecules were extracted from rabbit retina using methanol. Macromolecules in the retinal extract were removed by passing the extract through a dialysis membrane (Fig. 1A). This pool of retinal small molecules was incubated with recombinant MBP-NR2E3^LBD^. As control experiments, retinal small molecules were incubated without any protein or with recombinant MBP-NR2E1^LBD^. NR2E1, also known as TLX, is an orphan NR closely related to NR2E3^22,23^. Retinal small molecules bound to MBP-NR2E3^LBD^ were separated from unbound small molecules by a gel filtration chromatography. Retinal small molecules co-purified with MBP-NR2E3^LBD^ were identified by a reverse-phase HPLC-mass spectrometry (RP-HPLC–MS).

The RP-HPLC–MS result showed that biliverdin (observed m/z: 583.2556 (+ 1 charge) and 623.2480 (+ 1 charge); calculated exact mass: [M + H]^+^ C_33_H_35_N_4_O_6_^+^ 583.2557, [M + Na + H_2_O]^+^ C_33_H_36_N_4_O_7_Na^+^ 623.2482; error − 0.17 and − 0.23 ppm, respectively) was co-purified with MBP-NR2E3^LBD^ (Fig. 1B). These peaks were not observed when MBP-NR2E1^LBD^ was used instead of MBP-NR2E3^LBD^ (Fig. 1B). Commercial biliverdin showed a nearly identical RP-HPLC elution time and mass spectra (Fig. 1B and Figure S1 in the Supplementary Information available with the online version of this paper). This result suggests that biliverdin (Fig. 1C) might be a retinal small molecule specifically binding to NR2E3^LBD^.

**Figure 2:**
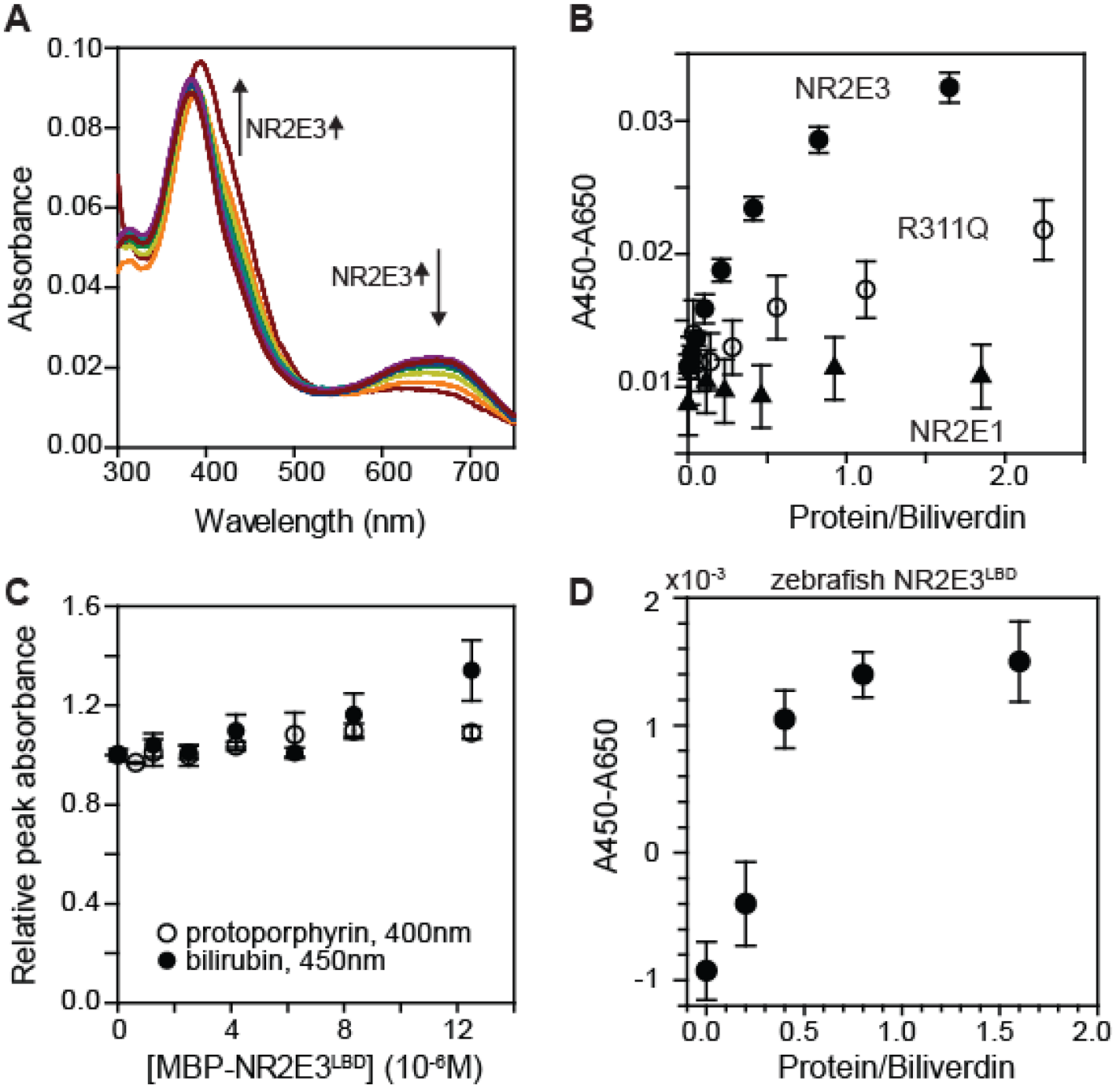
Biliverdin specifically binds to MBP-NR2E3^LBD^ in vitro. (**A**) Visible spectra of biliverdin (5 μM) in the absence and in the presence of varying amounts of MBP-NR2E3^LBD^. The blue peak shifts from 390 to 450 nm, while the red peak intensity diminishes upon binding to MBP-NR2E3^LBD^. (**B**) Biliverdin stoichiometrically bound to MBP-NR2E3^LBD^ (filled circle). MBP-NR2E3^LBD^ R311Q variant (open circle) showed a weaker binding to biliverdin (5 μM). MBP-NR2E1^LBD^ (triangle) did not show any evidence of binding to biliverdin. Averages and standard deviations (n = 3) are presented. (**C**) Effect of MBP-NR2E3^LBD^ on visible spectra of (filled circle) bilirubin and (open circle) protoporphyrin IX (average and standard deviations, n = 3). (**D**) Effect of MBP-zebrafish NR2E3^LBD^ to the visible spectrum of biliverdin (average and standard deviations, n = 3). The concentration of biliverdin was identical to the one used in Fig. 2A, B.

Next, we measured the binding of biliverdin to MBP-NR2E3^LBD^ in vitro (Fig. 2). Because biliverdin is a colored compound, the binding of biliverdin to MBP-NR2E3^LBD^ was measured by monitoring the visible spectrum of biliverdin while varying the concentration of MBP-NR2E3^LBD^ (Fig. 2A). Free biliverdin has two distinct absorption peaks—one in the blue region and the other in the red region. In the presence of MBP-NR2E3^LBD^, the blue absorption peak was shifted to a longer wavelength (from 390 to 430 nm) while the red peak intensity was decreased (Fig. 2A). This spectral change of biliverdin induced by MBP-NR2E3^LBD^ is distinct from the spectra of biliverdin bound to albumin^24^ or phytochrome proteins^25^. When small molecules from the biliverdin-MBP-NR2E3^LBD^ complex were recovered and analyzed by RP-HPLC-MS, the HPLC retention time and the mass-to-charge ratio of the recovered compound was identical to free biliverdin (Figure S2). This result indicates that the spectral change of biliverdin bound to MBP-NR2E3^LBD^ is reversible upon the removal of the protein. Biliverdin and NR2E3^LBD^ formed a stoichiometric complex. A plot of the blue absorbance peak intensity against the MBP-NR2E3^LBD^ concentration showed that biliverdin stoichiometrically bound to MBP-NR2E3^LBD^ with a K_D_ of 0.2 μM (Fig. 2B).

Next, we asked whether an Enhanced S-Cone Syndrome (ESCS) mutation affects biliverdin’s binding to NR2E3^LBD^. A blind-docking simulation of the biliverdin-NR2E3^LBD^ interaction suggested that the compound bound to a pocket adjacent to the R311 residue (Figure S3). We chose the R311Q mutation because this mutation is also a pathological mutation frequently found in ESCS patients (ClinVar accession number VCV000005532). We found that MBP-NR2E3^LBD^ R311Q variant was poorly bound to biliverdin (Fig. 2B, calculated *K*_D_ 9 μM). This result indicates that the R311 residue is necessary for biliverdin to bind to NR2E3^LBD^, and suggests that the interaction of biliverdin and NR2E3 might be relevant to the ESCS phenotype.

This interaction of biliverdin and MBP-NR2E3^LBD^ was specific. First, biliverdin did not show any evidence of binding to MBP-NR2E1^LBD^ at micromolar concentrations (Fig. 2B). The NR2E1^LBD^ protein sequence is ~ 48% identical to NR2E3^LBD^, and it is a closest neighbor of NR2E3 among members of the NR family proteins (Figure S3). Second, NR2E3^LBD^ distinguished biliverdin from similar compounds (Fig. 2C). The visible spectrum of bilirubin was little affected by MBP-NR2E3^LBD^ until the protein concentration reached nearly 10 μM. Unlike peroxisome proliferator-activated reporter alpha (PPARalpha), which is an NR binding to both bilirubin and biliverdin at micromolar concentrations^24^, NR2E3 is more specific to biliverdin. Considering that serum concentration of bilirubin is higher than that of biliverdin, this selectivity of NR2E3 toward biliverdin over bilirubin might be physiologically relevant. We also observed that protoporphyrin did not show any evidence of binding to MBP-NR2E3^LBD^ at micromolar concentrations (Fig. 2C). Taken together, these results indicated that the biliverdin is a specific ligand for NR2E3^LBD^ at least in vitro.

This interaction between biliverdin and NR2E3 might be conserved in other organisms. Zebrafish NR2E3^LBD^ is ~ 73% identical to human NR2E3^LBD^ at the amino acid sequence level (Figure S4). When the visible spectrum of biliverdin was measured varying the concentration of zebrafish NR2E3^LBD^ (MBP-DrNR2E3^LBD^), the spectrum of biliverdin showed changes similar to the one observed with human NR2E3^LBD^ (Fig. 2D). This result suggests that the binding of biliverdin may be conserved in other organisms.

### Biliverdin induced NR2E3-dependent reporter gene expression

**Figure 3:**
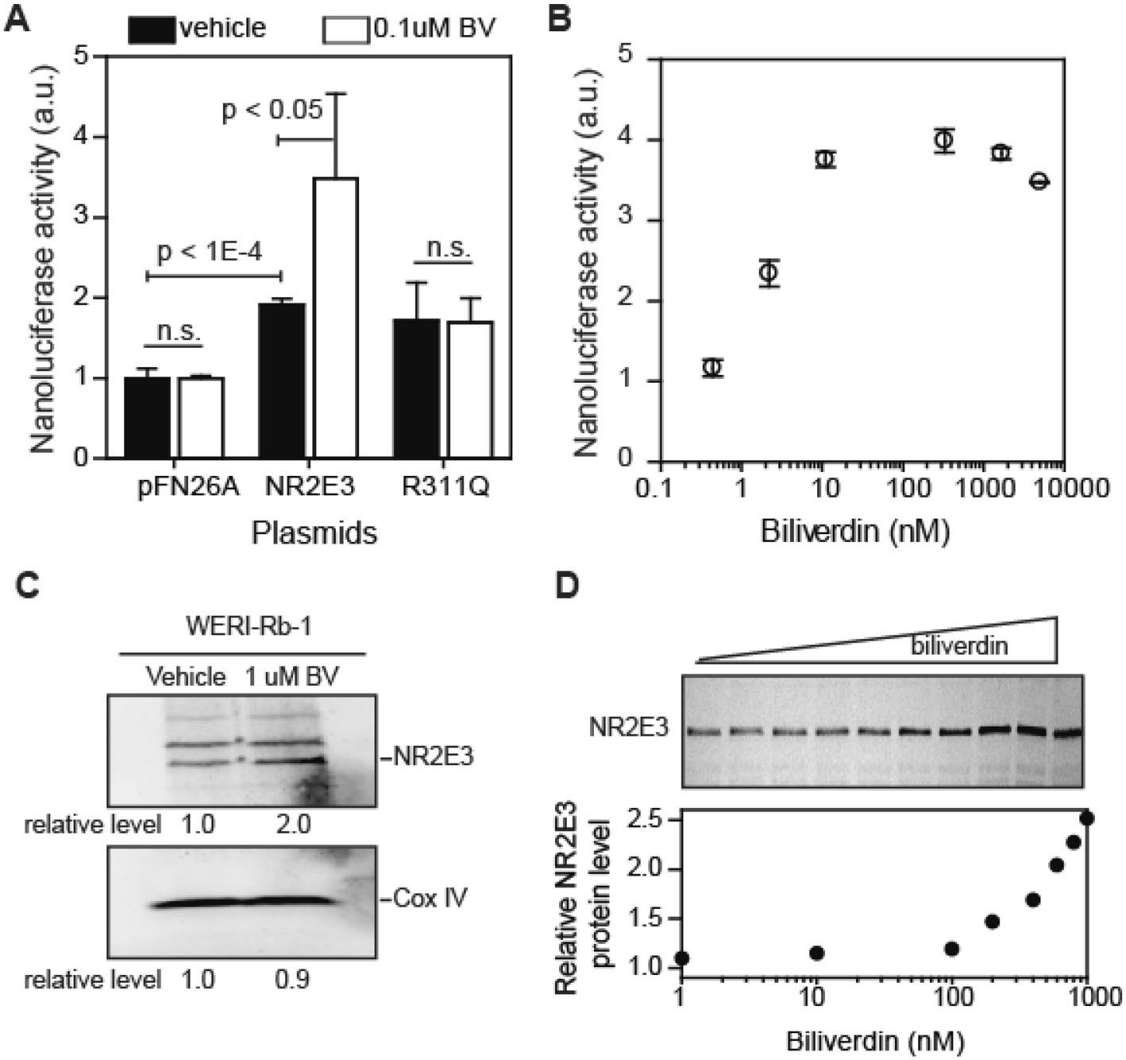
Biliverdin regulates NR2E3 in cells. (**A**, **B**) Reporter gene expression analysis in 293F cells. Cells were transfected with a reporter plasmid encoding nanoluciferase under the control of 9 × GAL^UAS^ and plasmids (pFN26A derivatives) encoding GAL^DBD^-NR2E3 fusion protein. A day after transfection, cells were treated with vehicle (0.1% DMSO) or biliverdin for 3 h. Nanoluciferase activity was measured using Vivazine substrate (average and standard deviations, n = 3). (**C**, **D**) Biliverdin increased NR2E3 protein level in WERI-Rb-1 cells. WERI-Rb-1 human retinoblastoma cells were incubated with vehicle (0.1% DMSO) or biliverdin (1 μM for **C** and varying concentrations for **D**) for 24 h. Western blot results (1:1000-fold diluted antibody) are shown. COX IV was used as a loading control. In Fig. 3C, different parts of the same gel are shown. Full-sized Western blot images are available in the Supplementary Information.

Next, we asked whether biliverdin affects transcriptional activity of NR2E3 in cells. For this purpose, we used a reporter gene expression in 293F cells. Because serum can contain variable amounts of biliverdin (0.9–6.5 µM total biliverdin, that includes free biliverdin and ones bound to blood proteins like albumin^26^), we used serum-free chemically defined medium in this experiment. We found that biliverdin (0.1 μM) significantly (*p* < 0.01) induced the NR2E3-dependent reporter gene expression by ~ threefold (Fig. 3A). At the tested concentration (0.1 μM), biliverdin only marginally affected the NR2E3 R311Q-dependent reporter gene expression, which reflects our in vitro results (Fig. 3A). When the experiment was repeated varying the concentration of biliverdin, the effective concentration at 50% of the maximum effect (EC_50_) was calculated to be 5 nM (Fig. 3B). At concentrations above 10 μM, biliverdin affected the viability of 293 cells. We thus carried out subsequent cellular and in vivo experiments at lower concentrations of biliverdin. These results indicated that biliverdin regulates NR2E3’s function in cells and that the loss of this interaction might be a reason behind the ESCS phenotype observed with the R311Q mutation.

Next, we asked whether biliverdin affects NR2E3 in cells naturally expressing NR2E3. We thus tested effects of biliverdin on WERI-Rb-1^27^ and Y79 retinoblastoma cells. Although retinoblastoma cells do not recapitulate all aspects of retinal photoreceptor cells, NR2E3 is expressed in retinoblastoma cells^28^ and regulates its own expression^29^. Our Western blot analysis of NR2E3 in retinoblastoma cells showed that biliverdin induced the level of NR2E3 protein by approximately twofold (2.0 ± 0.2 fold elevation by 1 μM biliverdin, n = 3; Fig. 3C, S5) in a dose-dependent manner (Fig. 3D, not repeated). Taken together with the reporter gene expression analysis, this result suggests that biliverdin regulates NR2E3 levels in cells.

### Biliverdin regulates photoreceptor development in zebrafish larvae

Because of the importance of NR2E3 in retinal photoreceptor cell development, we tested whether biliverdin is required for retinal photoreceptor cell development. Several reasons made zebrafish a good model system for this purpose^30–32^. First, zebrafish eyes are similar to humans^30^. Second, the necessity of NR2E3 in zebrafish photoreceptors has also been demonstrated^7^. Third, monitoring early retinal developmental processes is feasible with zebrafish embryos. Finally, delivery of small molecules to retina can be more readily achieved in zebrafish embryos than mammals^31^.

**Figure 4:**
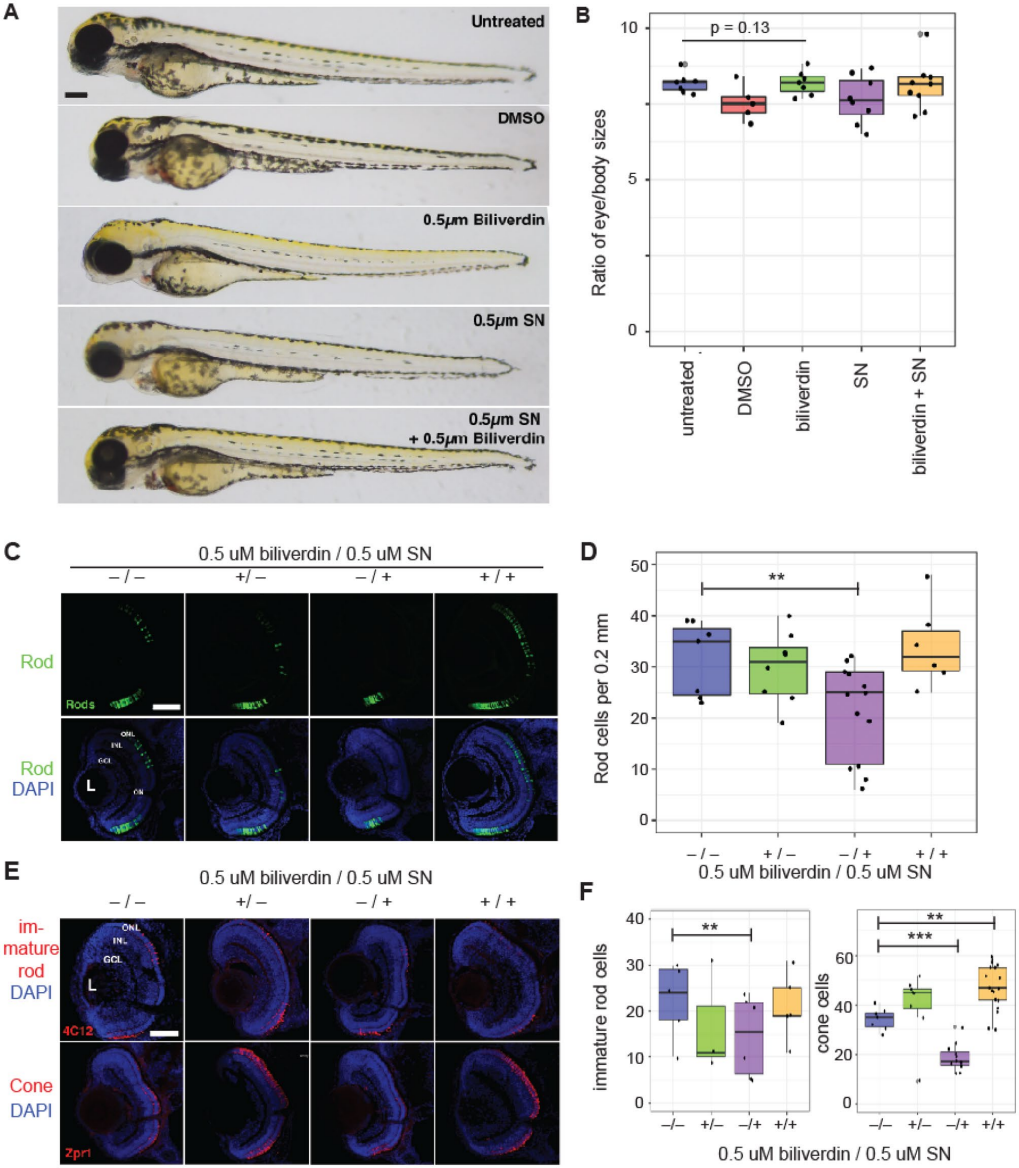
Biliverdin contributes to retinal development in zebrafish larvae. (**A**, **B**) Biliverdin and sulconazole at the tested concentrations (0.5 μM each) did not affect the overall development of zebrafish larvae. (**A**) Whole mount images of 3 dpf zebrafish larvae. (**B**) Quantification of the eye and body size ratios. (**C**) Fluorescence microscopic images of 3 dpf zebrafish larvae (from left to right) without any treatment, with 0.5 μM biliverdin, with 0.5 μM sulconazole nitrate, and with both biliverdin and sulconazole nitrate (top: rod photoreceptor cells, bottom: overlay of rod photoreceptor cells and DAPI-stained cells). (**D**) Quantification of rod photoreceptor cells in zebrafish larvae (3 dpf) grown in different conditions (**p = 0.00369). (**E**) Fluorescence microscopic images of zebrafish larvae (top) immunolabeled with the 4C12 antibody (which detect immature rod photoreceptors) and (bottom) red-green cones immunolabeled with the Zpr-1 antibody. Nuclei were counterstained with DAPI. (**F**) Quantification of immature rods and red-green cones (***p < 10^−5^). ONL, outer nuclear layer; INL, inner nuclear layer; GCL, ganglion cell layer. Scale bars, 0.1 mm in A; 0.05 mm in **C**, **E**.

Because there are multiple isoforms of HO isozymes and BVR isozymes in zebrafish, we used a pharmacological HO inhibitor to suppress biliverdin synthesis. Sulconazole nitrate (SN) inhibits HO isozymes in vitro and in vivo and would be suitable for this purpose^33^. At tested concentrations, neither SN nor exogenously supplied biliverdin caused observable developmental defects in zebrafish embryos (Fig. 4A, B). In contrast, fluorescence microscopic analysis of retinas from XOPS-GFP transgenic larvae (which express GFP specifically in rod photoreceptors) showed an approximate 30% decrease in the number of rod photoreceptor cells in SN treated larvae at 3 days post fertilization (dpf; *p* = 0.0172, untreated n = 7, SN-treated n = 13; Fig. 4C, D). This decreased rod cell population was also reported to occur in nr2e3-ko zebrafish larvae^7^. This reduced rod phenotype in SN-treated zebrafish larvae was alleviated by exogenously supplied biliverdin (Fig. 4C, D). This result indicates that the decreased rod cell population was not because of the accumulation of heme but because of loss of biliverdin or its downstream product.

Interestingly, the pharmacological inhibition of biliverdin synthesis showed broader impacts on photoreceptor cell development than were reported for the zebrafish nr2e3-ko. We found that the population of red-green cone photoreceptors (immunolabeled with the Zpr-1 antibody), was also decreased by sulconazole nitrate at 3 dpf (*p* < 0.001; for rods: untreated n = 5, sulconazole nitrate-treated n = 6; for cones: untreated n = 5, sulconazole nitrate-treated n = 11; Fig. 4E, F). Exogenously provided biliverdin increased the cone photoreceptor cell population (Fig. 4E, F), demonstrating that this phenotype was specific to the loss of biliverdin. A similar decrease of immature rod photoreceptors (immunolabeled with the 4C12 antibody) was also observed following SN exposure (Fig. 4E, F). Taken together, these results suggest that, in addition to the regulation of NR2E3 in rod precursors, biliverdin might be required for photoreceptor specification or differentiation upstream to the differentiation of rod photoreceptor cells, perhaps in pan-photoreceptor progenitors.

## Discussion

NR2E3 has been implicated in the development and the maintenance of retinal photoreceptor cells. Identifying its in vivo ligand is important in two aspects. First, the identity of an NR2E3 ligand provides a novel component contributing to retinal development. Second, this ligand can serve as a template to develop a reagent for translational therapeutic purposes.

Our results provide another piece of data connecting heme metabolism to retinal development. In addition to NR2E3, there are several NRs involved in retinal development ^34^. One NR playing an important role in retinal photoreceptor cell development and beyond is NR1D1 (also known as Rev-Erb alpha), which functions in concert with NR2E3 in retinal development while having an additional role in circadian rhythm. Heme itself is a ligand regulating NR1D1 and circadian rhythm^17,35,36^. In this study we show that biliverdin, the immediate downstream product of heme, regulates NR2E3, further suggesting a connection between heme metabolism and retinal photoreceptor transcription network. Our results suggest that inhibition of biliverdin synthesis results in a broader impact on zebrafish photoreceptor development than what was reported with nr2e3-ko animals; both rod and cone photoreceptors were reduced at 3 dpf in biliverdin-inhibited larvae, whereas only rods were affected at early stages in nr2e3-ko zebrafish, although the cones eventually showed signs of degeneration in adults^7^. Further analysis at later differential stage might be informative. We speculate that this expanded role for biliverdin in photoreceptor development may be due to additional binding to other NR proteins by biliverdin or its downstream metabolites in photoreceptor progenitors. Interestingly, no increase in short-wavelength cones was observed in the nr2e3-ko zebrafish study (in contrast to the mouse and human photoreceptor phenotypes resulting from loss of Nr2e3); since our zebrafish experiments only analyzed the numbers of red and green cones, it remains to be determined whether loss of biliverdin could also result in an increase in short-wavelength (blue and UV) cones in zebrafish.

Why biliverdin? We suspect that there might be a connection to light and/or oxidative stress response. As a light-absorbing molecule, biliverdin is used as a chromophore sensing red light in plants and some bacteria^25,37^. Our spectroscopic result, however, suggests that biliverdin-NR2E3 has a spectral property distinct from free biliverdin or plant phytochromes. This spectral property of biliverdin-NR2E3 makes it unlikely to sense red light as in plant phytochromes. Instead, it is possible that biliverdin-NR2E3 may sense blue light. In addition, biliverdin can function as a lipophilic redox signaling molecule. Excess heme can cause oxidative damage to light-exposed cells, and HO isozymes protect these cells from oxidative stress^38–41^. In general, this protective function of HO isozymes is believed due to the removal of excess hemes. By binding to biliverdin, the product of HO isozymes, NR2E3 may indirectly sense light and oxidative stress. Indeed, in non-retinal MCF-7 breast cancer cells, oxidative stress-causing benzopyrene reduced the expression of *NR2E3*, which is rescued by antioxidant^42^. In MCF-7 cells, NR2E3 regulates the expression of aryl hydrocarbon receptor, implying relevance to NR2E3 in oxidative stress response in these cells^43^. At this stage, it is not clear whether biliverdin-NR2E3 directly or indirectly senses light.

NR2E3 is expressed in the adult as well as the developing retina. Genetically overexpressing NR2E3 showed beneficial effects in several different retinitis pigmentosa models, suggesting that an increased NR2E3 activity can be beneficial^11^. We speculate that biliverdin or its derivatives might be useful in the development of reagents targeting retinitis pigmentosa.

## Methods

### Plasmids and proteins

Plasmids used in this study are summarized in the Supplementary Information Table S1 available with the online version of this paper. Plasmids constructed for this study were from GenScript and sequenced from both ends of inserts. Proteins were expressed and purified from BL21 (DE3) CodonPlus RILP cells (Agilent) harboring appropriate plasmids. For the purification of MBP-NR2E3^LBD^, cells were grown in 1-L Terrific Broth supplemented with 50 µg/mL ampicillin and 34 µg/mL chloramphenicol at 37 °C until OD_600_ became 1. Protein expression was induced by the addition of isopropylthio-beta-D-galactopyranoside (IPTG) to 0.5 mM, followed by an overnight incubation at 12 °C. Cells were harvested by centrifugation (5000 g for 20 min at 4 °C), and the pellet was resuspended in 20 mL buffer 1 (20 mM HEPES, pH 7.4, 150 mM NaCl, 0.5 mM tris(2-carboxyethyl)phosphine hydrochloride or TCEP, 10 mM imidazole, 0.01% IGEPAL CA-630) supplemented with 1 mM phenylmethylsulfonyl fluoride (PMSF). Cells were lysed by sonication and cleared by centrifugation (15,000 g for 20 min at 4 °C). The supernatant was loaded onto a 5 mL His-Trap column (Cytivia) pre-equilibrated in buffer 1. The column was washed with 50 mL buffer 1, and bound proteins were eluted with buffer 1 containing 250 mM imidazole. The eluted proteins were concentrated using Amicon Ultracel-15 (molecular weight cut off 10 kDa) and dialyzed several times against 20 mM HEPES, pH 7.4, 150 mM NaCl, 0.5 mM TCEP, 0.01% IGEPAL CA-630. MBP-NR2E1^LBD^ was also prepared using an identical method. Protein contents were analyzed by SDS-PAGE and Coomassie Blue staining. Protein concentrations were determined by BCA assay (when IGEPAL CA-630 was used) or by measuring A_280_ in denaturing conditions.

### Enrichment of protein-binding metabolite

Young rabbit retinas (Pel Freez, six retinas, 0.6 g wet weight) were placed in a mortar containing 70 mL methanol and dry ice. Tissues were ground with a pestle while adding dry ice to keep the temperature low. Debris was removed by centrifugation at 3000* g* for 10 min at − 10 °C. The supernatant was filtered through a 0.2 micron PES filter and through an Amicon Ultracel-15 (mwco 3 kDa) centrifugal filter at 4 °C. The filtrate was freeze-dried at − 80 °C and stored at − 80 °C for up to 4 weeks. MBP-NR2E3^LBD^ (12 µM, 1 mL) was passed through a HiPrep 26/10 Desalting column pre-equilibrated with buffer 2 (100 mM triethylamine bicarbonate, 10 mM 2-mercaptoethanol, 10% v/v methanol) and eluted with buffer 2 as an eluent. Dried metabolites were dissolved in 20 mL buffer 2, and any insoluble materials were removed by centrifugation at 3000* g* for 30 min at 4 °C. A 5-mL protein solution was mixed with an equal volume of metabolite solution, and the mixture was incubated at ambient temperature for 1 h. Controls (5 mL protein solution diluted with 5 mL buffer 2, 5 mL buffer mixed with 5 mL metabolite solution, and metabolites mixed with MBP-NR2E1^LBD^) were also prepared. These solutions were cooled at 4 °C and passed through a HiPrep 26/10 column pre-equilibrated in buffer 2 with a flow rate of 10 mL/min at 4 °C. Protein-containing fractions were collected and passed through Amicon Ultracel-15 (mwco 3 kDa) filters. Filters were washed once with methanol containing 0.1% formic acid to recover any residual metabolites tightly bound to proteins. Filtrates were dried at − 80 °C in vacuum, and dried metabolites were dissolved in 0.20 mL methanol containing 0.1% formic acid. A 50-µL portion of this solution was injected to a reverse-phase HPLC-ESI-qTOF mass spectrometer (column: Phenomenex 00B-4336-E0, Synergi Polar-RP 4 micron beads 50 × 4.6 mm column; injection volume 50 µL; flow rate: 0.3 mL/min; solvent A: water with 0.1% formic acid; solvent B: acetonitrile with 0.1% formic acid; gradient: 0–5 min 1% B, 5–25 min: 1–90% B, 25–30 min: 90% B, 30–35 min 90 to 1% B, 35–45 min 1% B; positive ion mode detection, 50–1700 m/z range, 1 Hz acquisition frequency, 350 °C drying gas temperature, 12 L/min 50 psig nitrogen drying gas). Data were acquired and analyzed using a MassHunter software package and MZedDB database.

### Visible spectroscopy

Solutions (0.20 mL, 20 mM HEPES, pH 7.4, 150 mM NaCl, 0.5 mM TCEP, 0–10 μM protein, typically 5 μM biliverdin, 1% DMSO, 0.01% IGEPAL CA-630) were placed in Greiner Bio-One UV-Star UV-transparent microplate or in a quartz microcuvette (Starna Cells). Solutions were incubated in dark for 30 min at 22 °C. Spectra were acquired at 22 °C using a Varioskan multi-mode microplate reader or Beckman DU640 spectrophotometer. After acquiring the visible spectrum, the sample was diluted with 9 volumes of methanol and subjected to a reverse phase HPLC-ESI-qTOF mass spectrometric analysis. For this purpose, 10 μL of this diluted sample was injected to a Phenomenex Synergi Polar RP column connected to HPLC and ESI-qTOF instrument (flow rate: 0.3 mL/min at room temperature; solvent A: water with 10 mM triethylammonium bicarbonate; solvent B: acetonitrile with 10 mM triethylammonium bicarbonate; 0–5 min: 5% B, 5–10 min: 5–15% B, 10–60 min: 15–40% B, 60–80 min: 40–95% B, 80–90 min: 95 to 5% B, 90–95 min: 5% B; negative ion mode; 100–3200 m/z range; 1 spectra/s).

### Reporter assays

Sulconazole nitrate was from Sigma-Aldrich (catalog number 1623681). It was dissolved in anhydrous DMSO, and 1 mM stock solutions were stored at − 20 °C in aliquots. It was diluted with anhydrous DMSO further when desired. 293F cells (Thermo Fisher R79007) were grown and maintained in FreeStyle 293 Expression Medium (Thermo Fisher 12–338-018) at 37 °C 5% CO_2_. Cells were transiently transfected with plasmids using FreeStyle Max reagent (Thermo Fisher 16,447,100). Transfected cells were then incubated in medium containing biliverdin and/or sulconazole nitrate (0–10 µM, 1% v/v DMSO; biliverdin concentrations above 10 µM hampered cell viability) for 3–24 h. Expression of nanoluciferase was measured by adding Nano-Glo Vivazine substrate (Promega N2580).

### Zebrafish

All zebrafish lines were bred and raised at 28.5 °C on a 14-h light: 10-h dark cycle. The Tg(XlRho:EGFP) transgenic line (XOPS:GFP), has been previously described, and was obtained from James Fadool (Florida State University, Tallahassee, FL, USA^44^). Zebrafish were bred, raised, and maintained in accordance with established protocols for zebrafish husbandry ^45^. Embryos were anesthetized with ethyl 3-aminobenzoate methanesulfonate salt (MS-222, Tricaine; Sigma-Aldrich Corp., St. Louis, MO). All animal procedures were carried out in accordance with guidelines established by the University of Kentucky Institutional Animal Care and Use Committee. Animal care and experimentation were also in accordance with the ARRIVE guidelines.

### Zebrafish treatment, cryosections, immunohistochemistry and cell counts

Embryos were generated from XOPS:GFP in-crosses and randomly subdivided into groups of 5 at 48 h post fertilization (hpf). Each group was placed in one of 5 treatments in fish water: untreated, 0.1% DMSO, 0.5 µM sulconazole nitrate, 0.5 µM biliverdin, and 0.5 µM sulconazole nitrate + 0.5 µM biliverdin. At 72 hpf, embryos were fixed in 4% paraformaldehyde overnight, then incubated in 10% followed by 30% sucrose at 4 °C. Sectioning and immunohistochemistry were conducted as previously described ^46^ and immunolabeled sections were imaged on either a Nikon inverted (Nikon Ti-U) or confocal microscope (Leica SP8, Leica). The following antibodies were used: anti-4C12 (immature and mature rod photoreceptors, mouse, 1:100, provided by James Fadool, Florida State University) and anti-Zpr1 (red/green cone photoreceptors, 1:20, mouse, ZIRC). Slides were incubated in 4′,6-diamidino-2-phenylindole (DAPI) to label nuclei (1:10,000 dilution, Sigma). Photoreceptor cells were quantified by counting individual cells and at least 5 embryos were used for each analysis, across 3 biological replicates. Statistics were conducted using an one-way ANOVA followed by post-hoc Tukey test using GraphPad software. *P*-values less than 0.05 were considered significant and are indicated by *, *p* < 0.01 is indicated by **, and *p* < 0.001 by ***. Boxplots were generated using R (version 3.6.2), R studio (version 1.2.5033), and ggplot2 package (version 1.2.5033; http://ggplot2.tidyverse.org)^47^.

## Supplementary Information

Supplementary Information.

## References

[1] Schorderet DF, Escher P (2009). NR2E3 mutations in enhanced S-cone sensitivity syndrome (ESCS), Goldmann-Favre syndrome (GFS), clumped pigmentary retinal degeneration (CPRD), and retinitis pigmentosa (RP). Hum. Mutat..

[2] Haider NB (2000). Mutation of a nuclear receptor gene, NR2E3, causes enhanced S cone syndrome, a disorder of retinal cell fate. Nat. Genet..

[3] Coppieters F (2007). Recurrent mutation in the first zinc finger of the orphan nuclear receptor NR2E3 causes autosomal dominant retinitis pigmentosa. Am. J. Hum. Genet..

[4] Hennig AK, Peng GH, Chen S (2008). Regulation of photoreceptor gene expression by Crx-associated transcription factor network. Brain Res..

[5] Peng GH, Ahmad O, Ahmad F, Liu J, Chen S (2005). The photoreceptor-specific nuclear receptor Nr2e3 interacts with Crx and exerts opposing effects on the transcription of rod versus cone genes. Hum. Mol. Genet..

[6] Chen J, Rattner A, Nathans J (2005). The rod photoreceptor-specific nuclear receptor Nr2e3 represses transcription of multiple cone-specific genes. J. Neurosci..

[7] Xie S (1865). Knockout of Nr2e3 prevents rod photoreceptor differentiation and leads to selective L-/M-cone photoreceptor degeneration in zebrafish. Biochim. Biophys. Acta Mol. Basis Dis..

[8] Martinez-De Luna RI, El-Hodiri HM (2007). The Xenopus ortholog of the nuclear hormone receptor Nr2e3 is primarily expressed in developing photoreceptors. Int. J. Dev. Biol..

[9] Choudhary M, Malek G (2016). Rethinking nuclear receptors as potential therapeutic targets for retinal diseases. J. Biomol. Screen.

[10] Moore SM, Skowronska-Krawczyk D, Chao DL (2020). Targeting of the NRL pathway as a therapeutic strategy to treat retinitis pigmentosa. J. Clin. Med..

[11] Li S (2020). Nr2e3 is a genetic modifier that rescues retinal degeneration and promotes homeostasis in multiple models of retinitis pigmentosa. Gene Ther..

[12] Nakamura PA (2017). Small molecule Photoregulin3 prevents retinal degeneration in the Rho(P23H) mouse model of retinitis pigmentosa. Elife.

[13] Evans RM, Mangelsdorf DJ (2014). Nuclear receptors, RXR, and the big bang. Cell.

[14] McDonnell, M. C. & Mohiuddin, S. S. in *StatPearls* (2021).

[15] Kutty RK (1995). Induction of heme oxygenase 1 in the retina by intense visible light: suppression by the antioxidant dimethylthiourea. Proc. Natl. Acad. Sci. USA.

[16] Maines MD (2005). New insights into biliverdin reductase functions: linking heme metabolism to cell signaling. Physiology.

[17] Raghuram S (2007). Identification of heme as the ligand for the orphan nuclear receptors REV-ERBalpha and REV-ERBbeta. Nat. Struct. Mol. Biol..

[18] Yin L (2007). Rev-erbalpha, a heme sensor that coordinates metabolic and circadian pathways. Science.

[19] Keller KE, Doctor ZM, Dwyer ZW, Lee YS (2014). SAICAR induces protein kinase activity of PKM2 that is necessary for sustained proliferative signaling of cancer cells. Mol. Cell.

[20] Keller KE, Tan IS, Lee YS (2012). SAICAR stimulates pyruvate kinase isoform M2 and promotes cancer cell survival in glucose-limited conditions. Science.

[21] Tan MH (2013). The crystal structure of the orphan nuclear receptor NR2E3/PNR ligand binding domain reveals a dimeric auto-repressed conformation. PLoS ONE.

[22] Sun G, Cui Q, Shi Y (2017). Nuclear receptor TLX in development and diseases. Curr Top Dev Biol.

[23] Yu RT (2000). The orphan nuclear receptor Tlx regulates Pax2 and is essential for vision. Proc. Natl. Acad. Sci. USA.

[24] Gordon DM, Hong SH, Kipp ZA, Hinds TD (2021). Identification of binding regions of bilirubin in the ligand-binding pocket of the peroxisome proliferator-activated receptor-A (PPARalpha). Molecules.

[25] Krahmer J, Ganpudi A, Abbas A, Romanowski A, Halliday KJ (2018). Phytochrome, carbon sensing, metabolism, and plant growth plasticity. Plant Physiol..

[26] Gafvels M (2009). A novel mutation in the biliverdin reductase-A gene combined with liver cirrhosis results in hyperbiliverdinaemia (green jaundice). Liver Int..

[27] McFall RC, Sery TW, Makadon M (1977). Characterization of a new continuous cell line derived from a human retinoblastoma. Cancer Res..

[28] Kobayashi M (1999). Identification of a photoreceptor cell-specific nuclear receptor. Proc. Natl. Acad. Sci. USA.

[29] Haider NB (2009). Nr2e3-directed transcriptional regulation of genes involved in photoreceptor development and cell-type specific phototransduction. Exp Eye Res.

[30] Angueyra JM, Kindt KS (2018). Leveraging zebrafish to study retinal degenerations. Front. Cell Dev. Biol..

[31] Chhetri J, Jacobson G, Gueven N (2014). Zebrafish–on the move towards ophthalmological research. Eye (Lond.).

[32] Thomas JL, Thummel R (2013). A novel light damage paradigm for use in retinal regeneration studies in adult zebrafish. J. Vis. Exp..

[33] Kinobe RT (2006). Inhibition of the enzymatic activity of heme oxygenases by azole-based antifungal drugs. J. Pharmacol. Exp. Ther..

[34] Forrest D, Swaroop A (2012). Minireview: the role of nuclear receptors in photoreceptor differentiation and disease. Mol. Endocrinol..

[35] Mollema NJ (2011). Nuclear receptor Rev-erb alpha (Nr1d1) functions in concert with Nr2e3 to regulate transcriptional networks in the retina. PLoS ONE.

[36] Yin L, Wu N, Lazar MA (2010). Nuclear receptor Rev-erbalpha: a heme receptor that coordinates circadian rhythm and metabolism. Nucl. Recept. Signal.

[37] Wang H (2015). Phytochrome signaling: time to tighten up the loose ends. Mol. Plant.

[38] Zhao J (2012). Heme oxygenase and ocular disease: a review of the literature. Curr. Eye Res..

[39] Castilho A (2012). Heme oxygenase-1 protects retinal endothelial cells against high glucose- and oxidative/nitrosative stress-induced toxicity. PLoS ONE.

[40] Sun MH (2007). Photoreceptor protection against light damage by AAV-mediated overexpression of heme oxygenase-1. Investig. Ophthalmol. Vis. Sci..

[41] Kutty RK (1995). Induction of heme oxygenase 1 in the retina by intense visible light: suppression by the antioxidant dimethylthiourea. Proc. Natl. Acad. Sci. U S A.

[42] Khanal T, Kim D, Johnson A, Choubey D, Kim K (2015). Deregulation of NR2E3, an orphan nuclear receptor, by benzo(a)pyrene-induced oxidative stress is associated with histone modification status change of the estrogen receptor gene promoter. Toxicol. Lett..

[43] Khanal T (2017). Loss of NR2E3 represses AHR by LSD1 reprogramming, is associated with poor prognosis in liver cancer. Sci. Rep..

[44] Fadool JM (2003). Development of a rod photoreceptor mosaic revealed in transgenic zebrafish. Dev. Biol..

[45] Westerfield M (2000). The Zebrafish Book: A Guide for the Laboratory Use of Zebrafish (Danio rerio), 4th.

[46] Wen W, Pillai-Kastoori L, Wilson SG, Morris AC (2015). Sox4 regulates choroid fissure closure by limiting Hedgehog signaling during ocular morphogenesis. Dev. Biol..

[47] Wickham H (2016). ggplot2: Elegant Graphics for Data Analysis.

