# Supplementary material for: Biliverdin regulates NR2E3 and zebrafish retinal photoreceptor development
Source: Sci Rep. 2022 May 4;12:7310. doi: 10.1038/s41598-022-11502-3 (PMC9068610; doi:10.1038/s41598-022-11502-3)
Supplement: Supplementary file 1 — Supplementary Information. [file 41598_2022_11502_MOESM1_ESM.pdf]

Supplementary Materials for

Biliverdin regulates NR2E3 and zebrafish retinal photoreceptor development

Blaine Connor[1#], Kayla Titialii-Torres[2#], Abigail E. Rockenhaus[3#], Samuel Passamonte[3], Ann C. Morris[2], and Young-Sam Lee[3*]

*Correspondence to: youngsam.lee@uky.edu

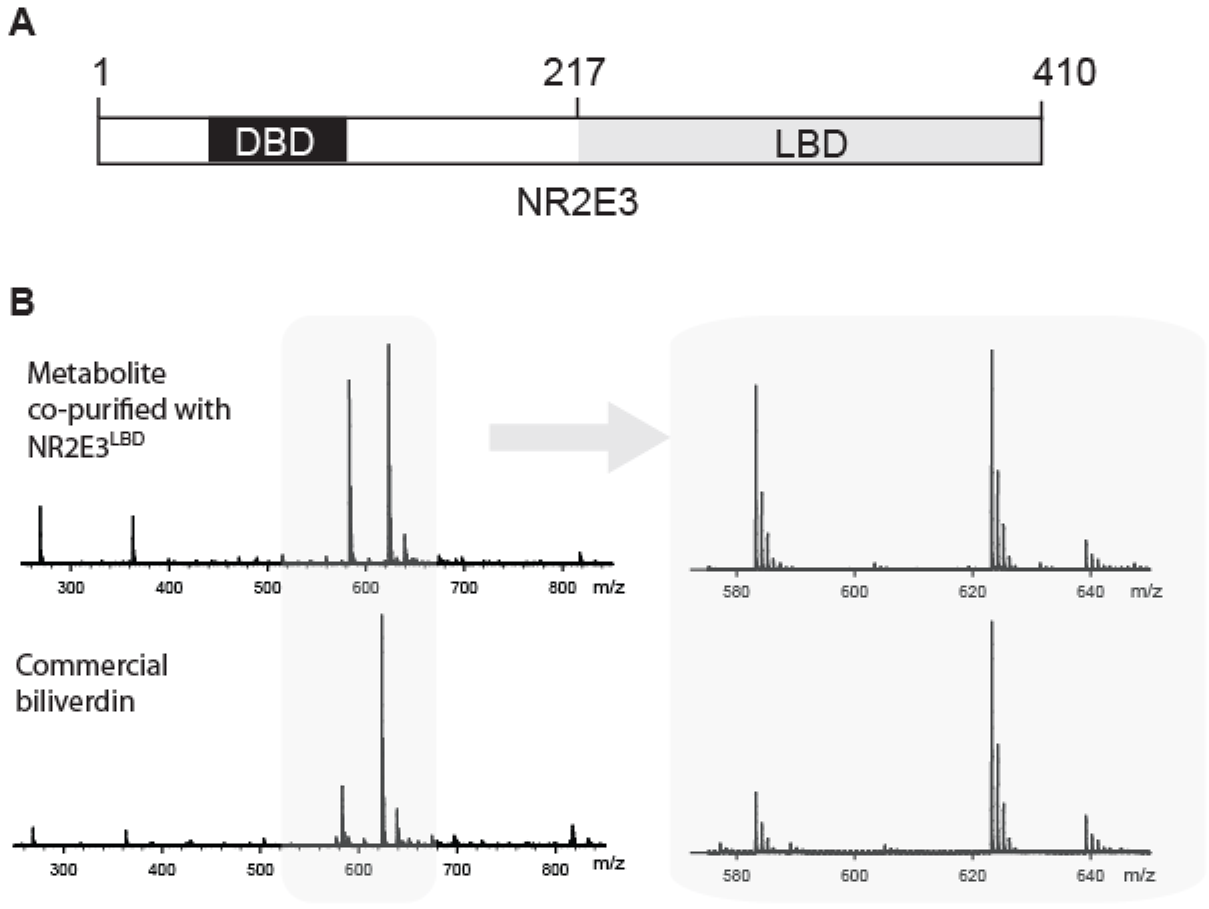

Supplementary Figure S1. Comparison of the metabolite co-purified with NR2E3^{LBD} with commercial biliverdin. (A) Domain architecture of human NR2E3. (B) Comparison of (top) metabolite co-purified with NR2E3^{LBD} and (bottom) commercial biliverdin.

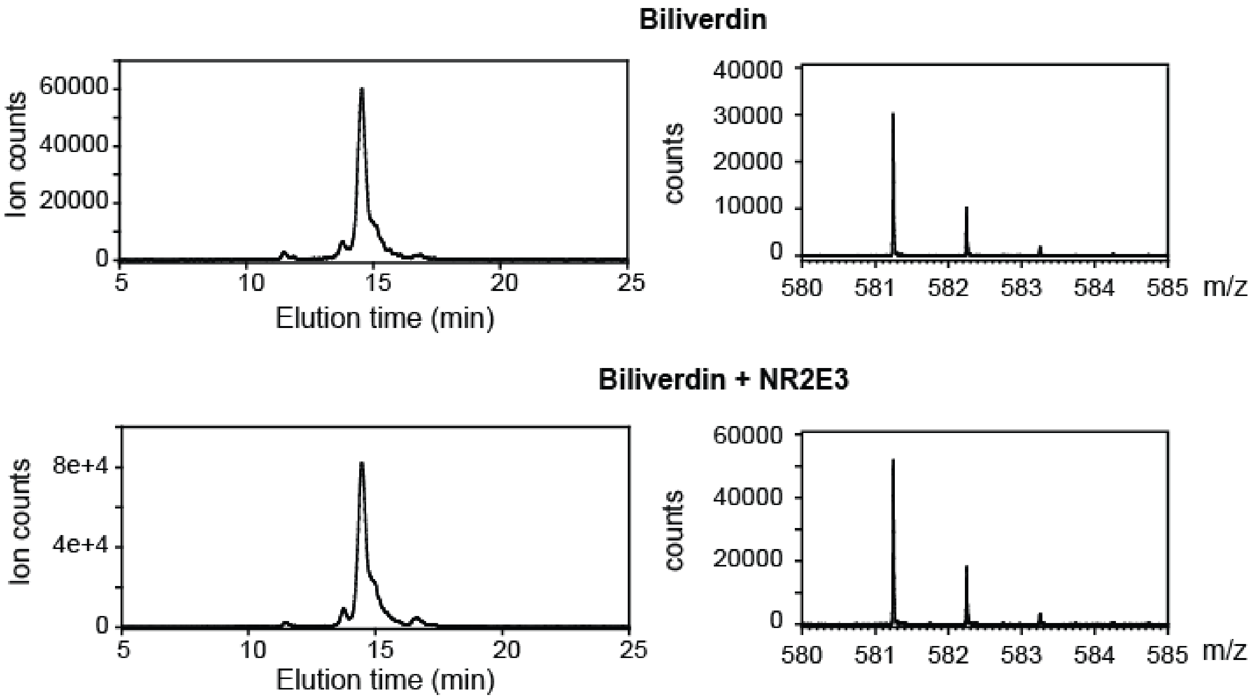

Supplementary Figure S2. Comparison of free biliverdin and the compound recovered from NR2E3^LBD^-bound biliverdin. (Left) extracted ion chromatograph of biliverdin (m/z 581.2400 for the M-H ion) and (right) mass spectra of (top) free biliverdin and (bottom) the compound recovered from the biliverdin-MBP-NR2E3^{LBD} complex.

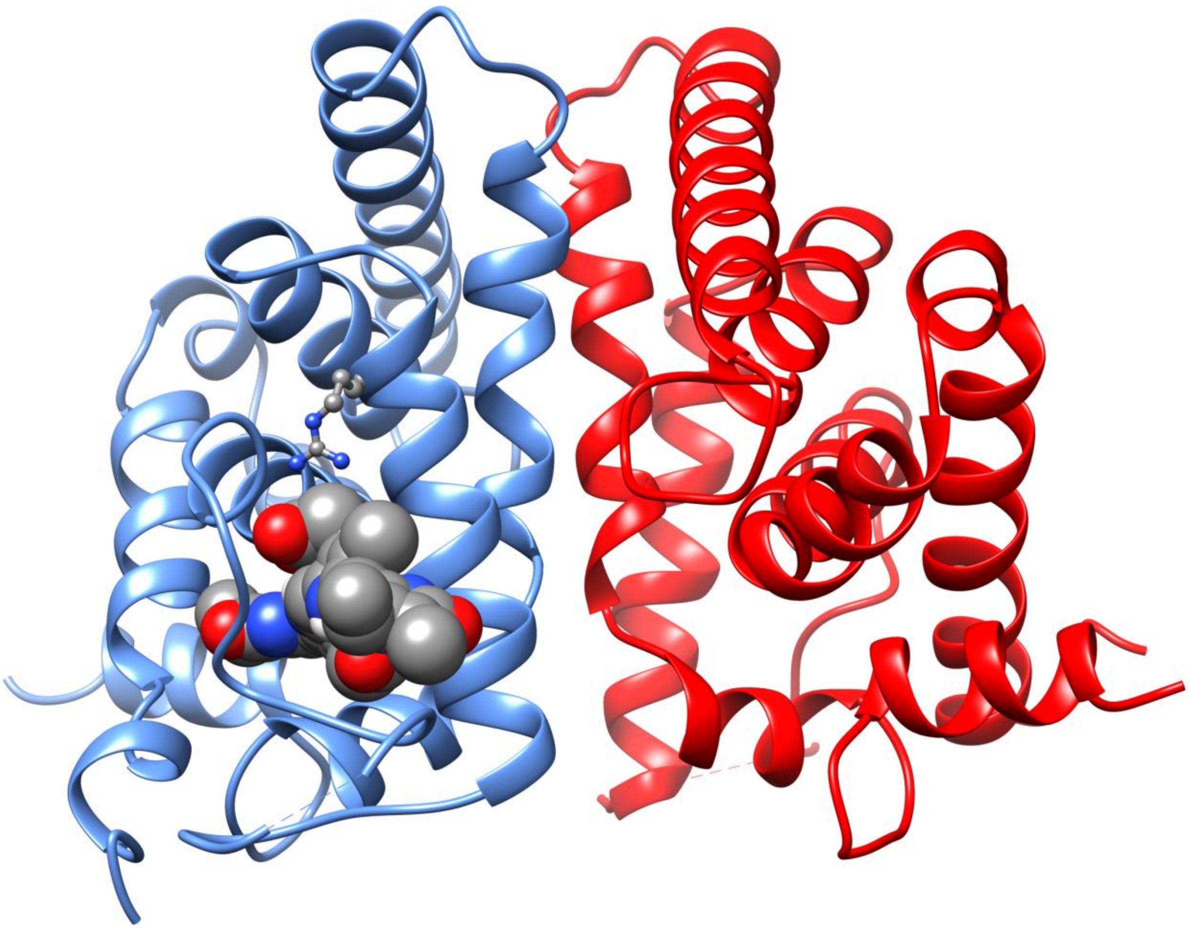

Supplementary Figure S3. A computational simulation of biliverdin's binding to a crystallographic structure of MBP-NR2E3^{LBD} homodimer. Biliverdin (PDB: BLA) was simulated to the crystallographic structure (PDB:4LOG, the MBP part of the structure was removed before the simulation) using Autodock Vina 4.0. The result was visualized using UCSF Chimera. Each chain of NR2E3^{LBD} is shown as red and blue ribbons. The lowest energy predicted binding mode of biliverdin is shown as spheres. An arginine 311 residue is shown as balls-and-sticks.

A

```
Human_NR2E3LBD      GLDSIHETSARLLFMAVKWAKNLPVFSSLPFRDQVILLEEAWSELFLLGAIQWSLPLDSC
Human_NR2E1LBD      -TESVCESAARLLFMSIKWAKSVPAFSTLSLQDQLMLLEDAWRELFVLGIAQWAIPVDAN
                      :*: *::******::****.:*.**:*.::**::***:** ***:**  **::*:*:

Human_NR2E3LBD      PLLAPPEASAAGGAQGRLTLASMETRVLQETISRFRALAVDPTEFACMKALVLFKP----
Human_NR2E1LBD      TLLAVSGMNGDNTDSQKLNKIISEIQALQEVVARFRQLRLDATEFACLKCIVTFKAVPTH
                    .*** .  .. .  . :*.    * :.***.::*** * :*.*****:*.:* **.

Human_NR2E3LBD      ---ETRGLKDPEHVEALQDQSQVMLSQHSKAHHPSQPVRFGKLLLLLPSLRFITAERIEL
Human_NR2E1LBD      SGSELRSFRNAAAIAALQDEAQLTLNSYIHTRYPTQPCRFGKLLLLLPALRSISPSTIEE
                       * *.:::.  : ****::*: *..: ::::*:** **********:** *:.. **

Human_NR2E3LBD      LFFRKTIGNTPMEKLLCDMFKN---
Human_NR2E1LBD      VFFKKTIGNVPITRLLSDMYKSSDI
                    :**:*****.*: :**.**:*.
```

B

```
Human_NR2E3LBD          GLDSIHETSARLLFMAVKWAKNLPVFSSLPFRDQVILLEEAWSELFLLGAIQWSLPLDSC
Zebrafish_NR2E3LBD      EPESVYETSARLLFMSVKWAKNLPVFSHLPFRDQVILLEEAWSELFLLCAIQWSLPLDNC
                          :*::*********:*********** ******************** *********.*

Human_NR2E3LBD          PLLAPPEASAAGGAQGRLTLASMETRVLQETISRFRALAVDPTEFACMKALVLFKPETRG
Zebrafish_NR2E3LBD      PLLSLPDLSPTG--QGKGSPSASDVRVLQEVFSRFKPLQVDPTEFACLKAIVLFKPETRG
                        ***: *: *.:*  **: : :: :.*****.:***:.* ********:**:*********

Human_NR2E3LBD          LKDPEHVEALQDQSQVMLSQHSKAHHPSQPVRFGKLLLLLPSLRFITAERIELLFFRKTI
Zebrafish_NR2E3LBD      LKDPEQVENLQDQSQVLLAQHIHTLYPSQVARFGRLLLLLPSLHFVSSERIEHLFFQRTI
                        *****:** *******:*:** :: :*** .***:********:*:::**** ***::**

Human_NR2E3LBD          GNTPMEKLLCDMFKN
Zebrafish_NR2E3LBD      GNTPMEKLLCDMFKN
                        ***************
```

Supplementary Figure S4. ClustalW sequence comparison of NR2E3^{LBD}. (**A**) A comparison of human NR2E3^{LBD} and NR2E1^{LBD} showed ~48% sequence identity. (**B**) A comparison of human and zebrafish NR2E3^{LBD} showed ~74% sequence identity.

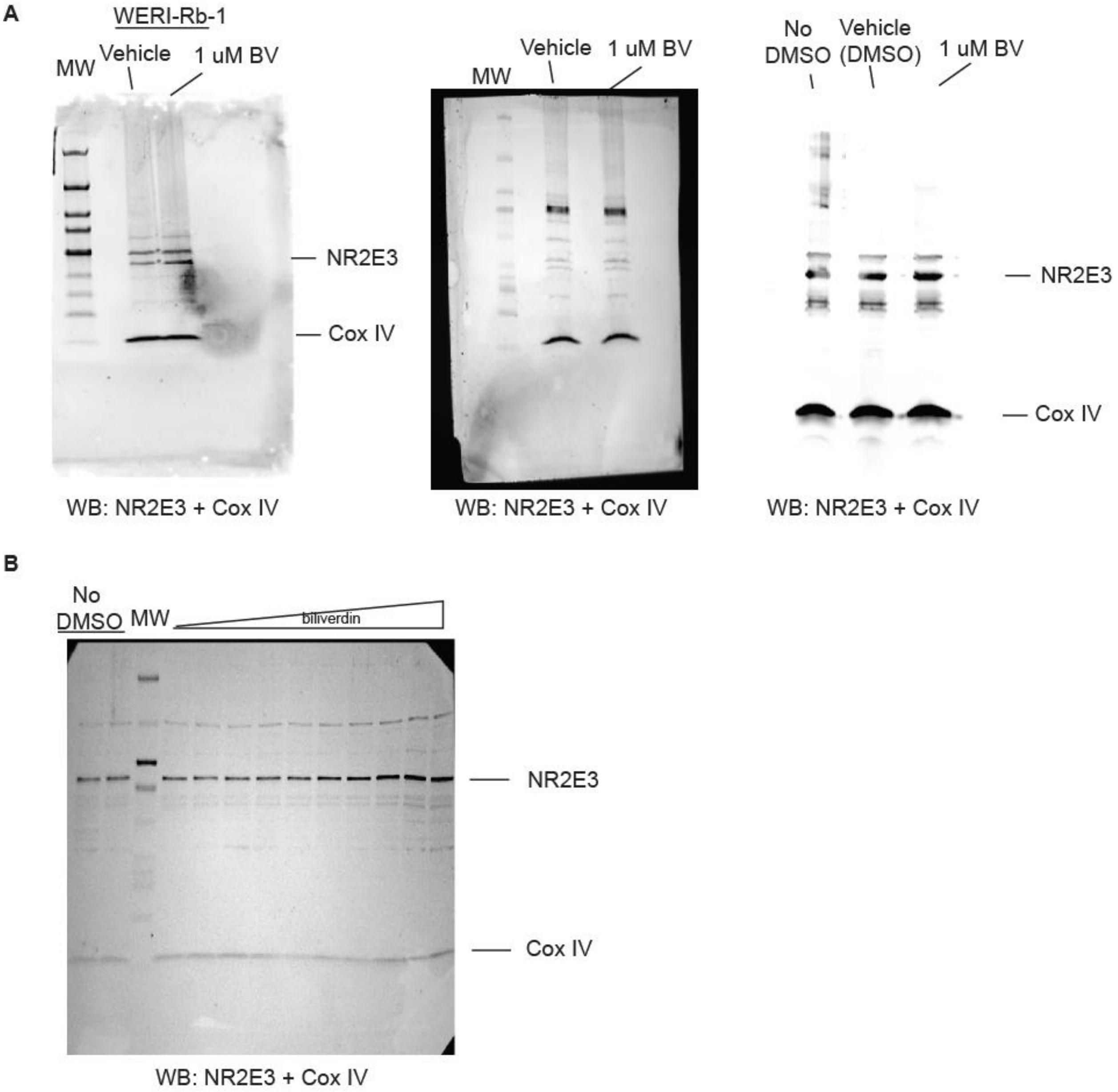

Supplementary Figure S5. Full-size Western blot images for ones presented in (A) figure 3C with two more repeats and (B) figure 3D, which was not repeated.

Reagents	Source	Identifier	Note/Ref.
**Antibodies**			
Cox IV rabbit monoclonal 3E11, HRP	Cell Signaling	5247S	1:1,000 for WB
Goat anti-mouse IgG, HRP	ThermoFisher	G-21040	1:1,000 for WB
NR2E3 antibody, polyclonal	Proteintech	14246-1-AP	1:10,000 for WB
4C12, monoclonal	Morris AC. et al. (2005)	ZDB-ATB-090506-2	1:100 dilution
Zpr-1 antibody	ZIRC	ZDB-ATB-081002-43	1:20 dilution
**Bacterial and Virus Strains**			
DH5α	Thermo Fisher	18265017	
BL21(DE3) Codon Plus RILP	Agilent	230280	
**Chemicals, Peptides, and Recombinant Proteins**			
Acetonitrile, LC-MS grade	Sigma-Aldrich	AX0156	
Ammonium acetate	Sigma-Aldrich	A7330	
Ampicillin	Thermo Fisher	11593027	
Bilirubin	Sigma-Aldrich	B4126	
Biliverdin	Sigma-Aldrich	30891	
Biliverdin	Cayman	19257	
Bovine serum albumin	Sigma-Aldrich	A7906	
Chloramphenicol	Thermo Fisher	BP904	
4',6-diamidino-2-phenylindole (DAPI)	Sigma-Aldrich	5.08741	
Dimethyl sulfoxide	Sigma-Aldrich	D2438	
Dithiothreitol	VWR	101170-432	
DPBS without calcium or magnesium	Thermo Fisher	14190250	
Fetal bovine serum	Thermo Fisher	A3160402	
Formic acid	Sigma-Aldrich	695076	
Freestyle 293 Expression medium	Thermo Fisher	12-338-018	
Freestyle MAX transfection reagent	Thermo Fisher	16-447-100	
Glycerol	VWR	97063-894	
Halt^{TM} protease and phosphatase inhibitors	Thermo Fisher	78444	
HEPES, 1M	Thermo Fisher	15630130	
Imidazole	Sigma-Aldrich	I5513	
Isopropylthio-beta-D-galactopyranoside	Thermo Fisher	AM9462	
Methanol, LC-MS grade	Sigma-Aldrich	MX0486	
Phenylmethylsulfonyl fluoride	Thermo-Fisher	36978	
Poly-D-lysine solution	Sigma-Aldrich	A-003-E	
Protoporphyrin IX disodium salt	Sigma-Aldrich	258385	
RIPA buffer	Thermo Fisher	89900	
RPMI-1640	Thermo Fisher	72400120	
Sodium chloride	Thermo Fisher	S271	
Sodium deoxycholate	Thermo Fisher	89904	
Sulconazole nitrate	Sigma-Aldrich	1623681	
TCEP	Thermo Fisher	77720	
Terrific Broth	Thermo Fisher	A1374301	
Tris-buffered saline, 20×	Thermo Fisher	28358	
Tris-buffered saline, Tween-20, 20×	Thermo Fisher	28360	
Water, LC-MS grade	Sigma-Aldrich	WX0001	
**Critical Commercial Assays**			
BCA Assay kit	Thermo Fisher	23250	
SuperSignal^{TM} West Femto	Thermo Fisher	34096	
**Experimental Models: Organisms/Strains**			

WERI-RB-1	ATCC	HTB169	
293-F	Thermo Fisher	R79007	
Danio rerio, Tg(XlRho: EGFP)	Obtained from Jim Fadool, Florida State University, Tallahassee, FL		1
**Recombinant DNA and Plasmids**			
pET21d-MAL-NR2E3$^{217-410}$-His$_6$	This work		
pET21d-MAL-NR2E3$^{217-410/R311Q}$-His$_6$	This work		
pET21d-MAL-NR2E1$^{182-385}$-His$_6$	This work		
pCDH-CMV-MCS- EF1α-GFP-Puro	System Biosciences	CD513B-1	
pCDH-GAL4^{UAS}-NlucP-EF1α-GFP-Puro	This work		
pFN26A	Promega	E1380	
pFN26A-NR2E3	This work		
pFN26A-NR2E3^{LBD}	This work		
pFN26A-NR2E3$^{\Delta LBD}$	This work		
pFN26A-NR2E3^{R311Q}	This work		

Supplementary Table S1. List of plasmids, antibodies, and cells used in this study.

Supplementary References

1. L. Yin *et al.*, Rev-erbalpha, a heme sensor that coordinates metabolic and circadian pathways. *Science* **318**, 1786 (2007).
2. Q. Qin *et al.*, In pursuit of synthetic modulators for the orphan retina-specific nuclear receptor NR2E3. *Journal of ocular pharmacology and therapeutics : the official journal of the Association for Ocular Pharmacology and Therapeutics* **29**, 298 (2013). PMC:3613967.
3. T. B. Acton *et al.*, Robotic cloning and Protein Production Platform of the Northeast Structural Genomics Consortium. *Methods in enzymology* **394**, 210 (2005).

1 Fadool, J. M. Development of a rod photoreceptor mosaic revealed in transgenic zebrafish. *Dev Biol* **258**, 277-290, doi:10.1016/s0012-1606(03)00125-8 (2003).
